# Provider and information technology operations staff perspectives on the feasibility of writing patient-generated health data into the electronic health record

**DOI:** 10.1093/jamiaopen/ooaf170

**Published:** 2026-02-15

**Authors:** Aman Saiju, Subiksha Umakanth, Anna Vaynrub, Romi Eli, Alissa Michel, Katherine D Crew, Rita Kukafka

**Affiliations:** Department of Population and Family Health, Mailman School of Public Health, Columbia University Irving Medical Center, New York, NY 10032, United States; Department of Bioethics, Columbia University School of Professional Studies, New York, NY 10032, United States; Department of Biomedical Informatics, Vagelos College of Physicians and Surgeons, Columbia University Irving Medical Center, New York, NY 10032, United States; Department of Medicine, Vagelos College of Physicians and Surgeons, Columbia University Irving Medical Center, New York, NY 10032, United States; Department of Biomedical Informatics, Vagelos College of Physicians and Surgeons, Columbia University Irving Medical Center, New York, NY 10032, United States; Department of Medicine, Vagelos College of Physicians and Surgeons, Columbia University Irving Medical Center, New York, NY 10032, United States; Department of Medicine, Vagelos College of Physicians and Surgeons, Columbia University Irving Medical Center, New York, NY 10032, United States; Herbert Irving Comprehensive Cancer Center, Columbia University Irving Medical Center, New York, NY 10032, United States; Department of Epidemiology, Mailman School of Public Health, Columbia University Irving Medical Center, New York, NY 10032, United States; Department of Biomedical Informatics, Vagelos College of Physicians and Surgeons, Columbia University Irving Medical Center, New York, NY 10032, United States; Herbert Irving Comprehensive Cancer Center, Columbia University Irving Medical Center, New York, NY 10032, United States; Department of Sociomedical Sciences, Mailman School of Public Health, Columbia University Irving Medical Center, New York, NY 10032, United States

**Keywords:** patient generated health data, fast healthcare interoperability resources, electronic health records, EHR editing, stakeholder analysis, Provider and IT perspectives

## Abstract

**Objectives:**

This study aims to develop a detailed understanding of provider and Information Technology (IT) operations staff experiences and attitudes regarding patients’ ability to edit their data. This includes understanding barriers to developing a process to write back data into the electronic health record (EHR) as well as a concrete set of recommendations on incorporating patient-generated data into the EHR.

**Materials and Methods:**

*RealRisks*, our team’s Fast Healthcare Interoperability Resources-compliant web-based patient decision aid, was utilized as an exemplar platform in which patients can access EHR data and review, correct, and contribute patient-derived data when specific elements are missing. An interview guide was developed and semi-structured interviews of 9 participants (physicians *n* = 4, IT operations staff *n* = 5) at Columbia University Irving Medical Center were carried out to understand the feasibility of writing back patient-entered edits into the EHR using the *RealRisks* decision aid.

**Results:**

Providers and IT operations staff reported varied knowledge of how patients interact with their data but collectively stated a need for increasing EHR accuracy that prioritizes provider-patient communication. Participants supported a write-back process and had specific suggestions for implementation mechanisms (such as the option to upload test results when submitting changes).

**Discussion:**

Providers and IT operations staff maintained that existing data management routes used for external data incorporation should be utilized, and that providers should screen edit requests to ensure EHR quality and accuracy.

**Conclusion:**

While participants felt a write-back of patient-derived data would be helpful, future studies should directly assess nursing staff and advanced practice providers as well as patient perspectives to ensure equity and efficacy.

## Introduction

Empowering patients is a key objective in public health, driving research and programmatic efforts to help patients better understand their health experiences and effectively communicate concerns to their providers. With the passage of the 21st Century Cures Act, the US Department of Health, and Human Services penalized information blocking, which is defined as restricting the passage and access of a patient’s information against the wishes of the patient.^1^ This includes the implementation of Information Technology (IT) such that Electronic Health Information (EHI) becomes directly inaccessible to the patient or causes difficulty for the patient to export the EHI to outside resources or other providers.^2^ The passage of this act places the patient as the owner and proprietor of their EHI, regardless of where that data is stored or what healthcare infrastructure has access to it.

Inherent to this ownership is the ability for patients to exert control over their EHI, including both access to and editing of their data. Editing EHI becomes particularly crucial given the high frequency of errors within a patient’s Electronic Health Record (EHR), with around 95% of patients experiencing discrepancies between the EHR and self-reported data.^3^ For these patients, self-reported data sources were around 90% accurate to their actual health experience, compared to an 80% accurate EHR.^3^ In addition to errors, patients health behaviors are susceptible to change over time, including smoking status, alcohol use, contraception, exercise, and more. Although this data may not have been entered into a patient’s EHR erroneously, it may be out of date, introducing significant imprecision in the clinically relevant information a provider would be referencing. Given such significant lapses in accuracy, the evolving landscape of healthcare demands that patients can easily update their data, including demographics such as race/ethnicity, family history of disease, health behaviors, and more.

Despite the high occurrence of EHR errors and subsequent patient detection, relatively few patients currently attempt to correct their EHI. This gap may be due to a low perceived likelihood of efficacy or inadequate knowledge about methods to change patient records.^4^^,^^5^ Existing methods of editing are time consuming and difficult, usually requiring an amendment request via a patient portal, or in the case of a patient preferring an off-line option, an in-person visit, phone call, or mail/fax from a patient to initiate the process and obtain requisite amendment request forms. Upon submission of these requests, the patient’s physician is then contacted to discuss the specific appeal, with the patient being notified of the results (approval or denial of the amendment) via their portal or by mail.^4^ This significant logistical and bureaucratic effort stymies close communication between the provider and patient, rendering efficient amendment inaccessible.

Furthermore, a prior study of patient-initiated amendment requests at the University of Michigan found that only 49.7% of the requests assessed were approved despite 77.8% of the requests being made to rectify incorrect information.^4^ The problem is thus 2-fold—the traditional methods of requesting amendments are inadequate, and even if a patient makes them, they are often declined for being unnecessary changes, inaccurate, or not having been made by the institution in question.^4^ As patient-initiated amendments utilize patients as the fundamental source of data, they are considered patient-generated health data (PGHD), as opposed to data generated by a provider’s perception of the patient’s health information (which forms the bulk of information within a patient’s EHR). Despite perceptions that data inputted by patients in a patient-initiated amendment is of poor quality or dilutes the accuracy of the EHR, PGHD has been shown to improve the accuracy and completeness of EHRs by filling information gaps, be more accurate for certain types of data elements, and can also improve patient health awareness and patient/provider communication, provided that providers can effectively interact with and become involved with PGHD.^6^

Technological advancements have been central to patient empowerment by increasing access to care and information. However, despite this increased connectivity, patient control and communication are still lacking.^7^ To this end, new highly integrated and visible mechanisms of incorporating patient feedback are critical.

### The *RealRisks* decision aid as a potential mechanism for editing

To examine the feasibility of diversifying the methods through which a patient can edit their EHR, we sought to utilize an exemplar platform that is external to the EHR but can still access patient EHI, enabling us to explore both the technical and workflow requirements of editing interfaces. Kukafka et al. have developed such a tool in the online decision aid called *RealRisks,* which helps patients assess their breast cancer risk, create a personalized risk profile, and generate action plans to reduce risk.^8^ This tool provides education about risk-aligned preventative services and informs interventions the patient may benefit from, such as chemoprevention and genetic testing. As part of developing their personalized risk assessment, users log into their patient portal from within *RealRisks* and, using Fast Healthcare Interoperability Resources compliant methods, can pull relevant data from their EHR. The patient can then edit and add missing data elements, including demographics, family history, past procedures, and imaging or biopsy results, creating a more complete and correct data set to be used by *RealRisks* in populating their risk assessment model.^8^ However, these additions and edits to a user’s PHI are not written back into the EHR but instead are maintained solely within the *RealRisks* platform. A write-back feature for *RealRisks* would be valuable in providing patients with the most comprehensive options for editing their EHI, enhancing user convenience and serving as a proof of concept for writing back into the EHR. Given this goal, we aimed to gather exploratory information from participants (including providers and IT operations staff) to understand potential barriers and ideal approaches for implementation.

## Methods

### Setting and recruitment

Study data were derived from interviews with 9 participants, including 4 providers (medical doctors) and 5 IT operations staff members (including informaticians and IT staff) from the Columbia University Irving Medical Center (CUIMC) in New York, NY, United States.

Participants were recruited using snowball sampling based on their background in patient health data management. Interviews were conducted to assess perspectives on the *RealRisks* decision aid in general and to understand the feasibility and ideal methodology of an EHR write-back process. Data were collected and analyzed by researchers at the Vagelos College of Physicians & Surgeons at CUIMC and the Mailman School of Public Health at CUIMC.

### Data collection

All 9 interviews were semi-structured and were conducted in English via video call by our lead researcher, who has received masters-level training in conducting qualitative research interviews. An interview guide was developed by our lead researcher and submitted for multiple rounds of review and editing by the research team at a weekly meeting. The flexibility afforded by the semi-structured nature of the interview guide allowed for greater ability to achieve thematic saturation during and across interviews. The interview guide sought to ascertain current practices through which patients access and edit their data, general feedback regarding the *RealRisks* tool, general perspectives regarding EHR write-back, and attitudes regarding potential methods of incorporating an EHR write-back into *RealRisks*. All 9 interviews were completed within a span of 6 months between November 2024 and April 2025, and interviews ranged from 27 to 62 minutes (with an average length of 43.6 minutes). Interviews were completed and recorded using Zoom. Recorded video files were promptly deleted, while the audio file was transcribed. Interview recordings and transcriptions were assigned a code number and separated from participants’ identifying information, with the code index file kept as an encrypted data file within the research team’s secure drive.

### Data analysis

**Figure 1.**
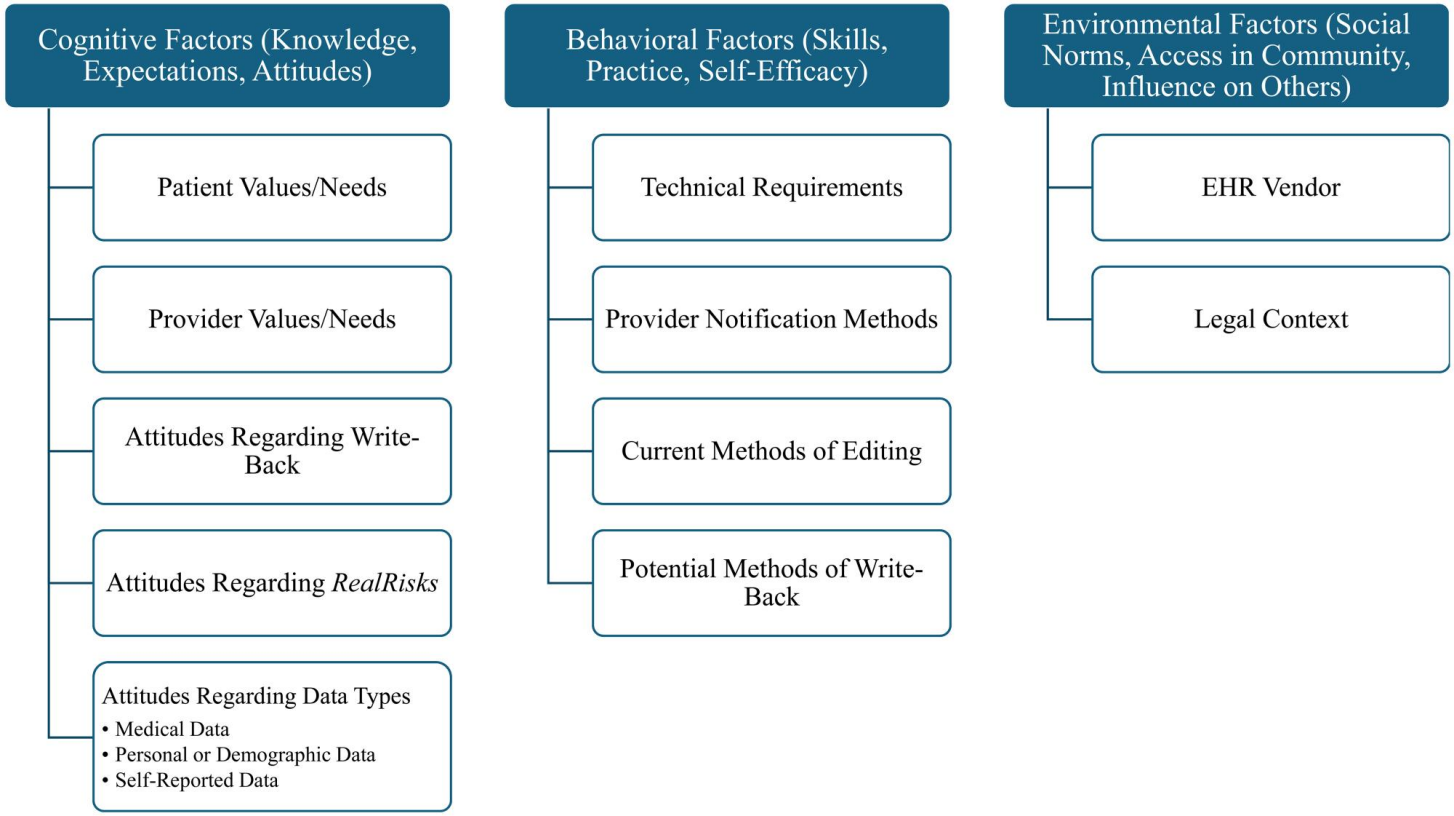
Schematic illustrating the organization of deductively derived codes developed from the 3 major constructs (column headings) of Social Cognitive Theory.

A codebook was generated based on a conceptual framework that applied Social Cognitive Theory.^9^^,^^10^ Codes were developed to assess behavioral, environmental, and cognitive factors, constructs within the theory that would contribute to generating participant perspectives and behaviors using a write-back method (Figure 1).

Our research team reviewed initial drafts of the codebook for feedback to ensure that the codes were neutral and addressed the entire scope of perspectives and relevant information present in the transcripts. Coding was completed using the Dedoose qualitative analysis software. In the case where novel codes were created partway through the coding process, memos were used as placeholders for pseudocodes. Once a formal code was developed, the transcripts were recoded to incorporate the new code.^11^ To maximize the consistency of the coding process, all coding was carried out by our lead researcher as a single coder. To determine the accuracy of coding and ensure that our lead researcher’s approach to coding remained consistent throughout the analysis period, an Intracoder Agreement (ICA) score was calculated using Dedoose by comparing the coding of one transcript by our lead researcher at 2 time points, 1 month apart. ICA was determined to be 0.87, meeting the generally accepted threshold of 0.8.^12^

Themes were established by assessing excerpts that contained key code overlaps (as identified by the Dedoose software). Quotes were grouped by concept (such as participant perceptions of patient agency and data-editing methodology, participant perceptions of institutional barriers, etc.) within a data display and arrayed along a positive/negative/neutral valence principle to highlight the variety of perspectives regarding each prevailing concept or idea. A summary was created for each group of quotes, and summaries across the valence principle were then used to derive various themes deductively. An example of the Data Display’s organization is shown in Table 1.

**Table 1.** Example organization of data display.

Valence principle	Positive	Negative	Neutral
**Deductively derived theme**			
Overarching concept	**Summary Idea** [Transcript Excerpt] [Transcript Excerpt]	**Summary Idea** [Transcript Excerpt] [Transcript Excerpt]	**Summary Idea** [Transcript Excerpt] [Transcript Excerpt]
Overarching concept	**Summary Idea** [Transcript Excerpt] [Transcript Excerpt]	**Summary Idea** [Transcript Excerpt] [Transcript Excerpt]	**Summary Idea** [Transcript Excerpt] [Transcript Excerpt]
**Deductively derived theme**			
Overarching concept	**Summary Idea** [Transcript Excerpt] [Transcript Excerpt]	**Summary Idea** [Transcript Excerpt] [Transcript Excerpt]	**Summary Idea** [Transcript Excerpt] [Transcript Excerpt]

## Results

As shown in Table 2, of the 9 participants interviewed, 4 were categorized as providers, and 5 were categorized as IT operations staff. However, 2 participants had experience as both providers and IT operations staff, although one self-identified as a provider and one self-identified as IT operations staff (despite having nursing experience) based on their current position. One provider also practices across both Emergency Medicine and Pediatrics.

**Table 2.** Participant demographics.

Participants (*n*)	9
Average age (provided by *n* = 8)	49
Race	
White	7
Hispanic or Latino	1
Not Hispanic or Latino	6
Asian	2
Gender	
Male	5
Female	4
Participant status	
Healthcare provider	4
Internal medicine	2
Pediatrics	1
Emergency medicine	1
Emergency medicine/pediatrics	1
IT operations staff	5

Participant perceptions on various themes were correlated with the valence principle defined in the Data Display. These themes identified included current methods of interaction with patient data, methods of writing-back, and considerations for application (Table 3).

**Table 3.** Summarization of themes and ideas deductively generated from participant quotes.

Theme	Summary ideas
Current Methods of Interaction with Patient Data	Participants are aware of how patients can utilize the Patient Portal to access their data.
	IT operations staff also discussed requesting physical copies via the HIM Office.
	Providers identified both the provider and HIM office as a method to request edits, but the current paradigm requires EHR edit requests to be approved by a provider. Direct methods, such as calling the HIM office, were also mentioned.
	IT operations staff stated that changes to certain forms of data (ie, DOB) had to go through the HIM office.
	Participants across the board felt that patients are interested in directly interacting with their data.
	IT operations staff stated that EHR data is currently flawed, especially resulting from data not being transferred to the new EHR during the recent adoption of EPIC.
Methods of Writing-back	Participants felt positive about reintroducing patient data into the EHR similar to how external data is brought in.
	Participants felt that providers should be approving requests for modification made to medical data. Demographic and personal data should be reported to the HIM office or privacy officer.
	Participants explicitly expressed their discomfort with allowing patients to overwrite existing data.
Considerations for Application	Providers have limited time, so implementation should not overburden them. IT operations staff felt that expanding existing workflows for write-back would not significantly increase provider workload.
	Write-back processes should be provided to patients in alignment with the timing of providers interacting with that patient’s data.
	Providers stated that data should be incorporated into the EHR in the same data domains that it would natively be found. Patients should be able to submit PDFs or other forms that verify requests for updates.
	Participants stated that their colleagues and coworkers would be open to implementing a write-back process, given the positive impact on patient engagement and interaction.
	IT operations staff raised concerns about whether a write-back would be well supported given that it requires trust in patient-entered data.
	Participants expressed concerns regarding the legal implications of a write-back process, given that data entered into the EHR is medical-legal information.

### Theme 1: Current methods of interaction with patient data

An important aspect of understanding the potential need for a write-back process included gauging methodologies through which patients can currently interact with their data. IT operations staff and providers cited the patient portal as the primary mechanism for patients to access and view their data (Table 4, Quote 1). However, only IT operations staff members also discussed physical copy requests, a process managed by the Health Information Management (HIM) Office (Table 4, Quote 2). The HIM office was stated to be the body responsible for carrying out amendments, with the ability to enter the EHR to modify the EHI itself. This pertained in particular to personal data, although providers could also request changes to medical data that had been previously saved (such as from previous visits or appointments).

**Table 4.** Participant quotes regarding current methods of interaction with patient data.

Quote #	Topic	Quote
1	Provider discussing the mechanism through which patients can access their data.	*Yeah, there’s a patient portal called MyChart or Connect, depending on if you are NYP or Columbia and patients can see large amounts of their data in that context, yeah. Excerpt P1 (line 19).*
2	IT operations staff member discussing mechanisms through which patients request physical copies of patient data.	*[patients] can actually sign an online authorization form, or paper form, for a release of information request and we can mail out those records to them, like a paper copy, email, fax or sometimes they ask for the records to be stored on a CD…I would say [patients] mostly [use] Connect. So, they will go onto the patient portal and look for that information. Excerpt P5 (lines 33-40).*
3	Provider discussing methods through which patients request data.	*[Connect] is the only way they can directly access that I know of. I know recently we have a way where you can sort of request the front desk to send patients a letter with their like recent lab results or certain information because we knew not everybody was on the portal, so we’re trying to get better at like sharing data with all of our patients; not just the people on the portal. Otherwise, yeah, I would imagine people can go to like medical records and ask for their data that way. Excerpt P1 (lines 23-27).*
4	Provider discussing the current workflow for editing patient data.	*A clinician isn’t going to refuse to put that information into the EHR. That’s not going to happen. What happens is, the encounter is closed, your clinic visit has ended, your hospitalization is over, whatever it may be, and you later see your EHR, and your notes and you say oh, this is wrong, didn’t do that. It’s very hard for a clinician to open what’s called an EHR encounter and change the information in there, so you usually have to go to health information management at the hospital and get them to change it, but they’re not the clinician so they don’t want to be changing the clinical data, so you get in this weird loop. So, if it’s during the encounter, it’s usually not a problem, as long as you’re a patient who can advocate for themselves, you know, you can make sure that happens. There can be issues there. It’s when it’s after the encounter and the error is noticed. Excerpt P7 (lines 210-221).*
5	IT operations staff member discussing workflow for editing medical data.	*You can see doctor’s notes and lab values and demographics, as you mentioned, yeah, so those are all inputted into the EHR by various different people and staff members. If something’s wrong in a note, and the note was written by a physician, then you would have to actually reach out to that physician to do an amendment to the note. Excerpt P5 (lines 53-54).*
6	IT operations staff member discussing patient desire for the ability to apply quality control to their data.	*We’re getting a lot of requests. Patients are reviewing their data and so they’re requesting those patient amendment requests… the reason being is, as you know, we do share information, medical records, through the health system through Epic, so if they sign off on the [Health Information Exchange] form, they want to make sure that the correct information is going to, [other hospitals], so they want to make sure if the information is going back and forth that it is correct. Excerpt P5, lines 50, 57-58.*
7	IT operations staff member discussing current patient engagement in editing their data.	*I think that we try to get them to [edit] and review as much as possible. I think the data shows that most patients don’t look to do that, they are more concerned with getting at their clinical data than they are worried about all of the other things…Sure, there are those who are vested in QAing the doctor’s note, which would have a lot of information in it, but yet, most just want to read what the doctor said or share what the doctor said with the next provider or family member or something like that. Excerpt P3 (30-43).*
8	Provider discussing current issue with data editing workflow.	*the risk is that what the patient requests gets ignored…It sort of sits there perpetually as a request if nobody addresses it. Excerpt P1 (lines 41 and 45).*

Operations staff suggested that patients were increasingly looking to utilize online resources, given their accessibility, but that some patients relied on physical methods due to low technological literacy or lack of technological resources. Compared to IT operations staff, providers did not have a robust technical knowledge of the HIM office’s processes (Table 4, Quote 3).

In addition to accessing data, participants were also asked about mechanisms currently offered to patients to edit their data. All participants identified outreach to the HIM office or provider as a method to request modifications, corrections, or edits to data. Providers suggested the patient portal as the method through which these requests would occur, including via direct in-basket messaging and suggesting appointment check-in as an effective touchpoint. However, IT operations staff also stressed the need for HIM office intervention to ensure that the data is updated effectively and efficiently within the current paradigm. A provider pointed out this siloed nature of the current patient data workflow as a potential problem (Table 4, Quote 4).

Despite the discrepancy between provider and IT operations staff understanding of patient data interaction, all participants stated that requests for changes to *medical* data currently must be approved by a provider (Table 4, Quote 5). Certain special forms of *identifying* data (ie, DOB, legal name) were said to require additional approval and verification by the HIM office or the institution’s privacy officer if any change is necessary. The privacy officer’s role was stated to be ensuring that any changes made were done so in a method compliant with confidentiality and information privacy regulations. They were thus often included in the approval process for any requested amendments.

All participants expressed that patients are interested in directly interacting with their data. IT operations staff stated that EHR data is flawed and that patients will likely need or want to update their data and health information (Table 4, Quote 6). However, one staff member cited that although the patient portal enables patients to read and access their notes and medical information, patients are not using all available resources. One operations staff member stated that although increasing the involvement of patients in their own data management process seems to be an institutional mission, patients are not effectively engaging in editing data (Table 4, Quote 7). One provider warned that even if patients request changes, requests may not be addressed (Table 4, Quote 8). Based on this observation, increasing patient access and control over their data by granting them the ability to carry out quality control was discussed as an important process moving forward.

### Theme 2: Methods of writing-back

Throughout the interviews, several options for implementing a write-back emerged as points of discussion by participants. These were classified as (1) a direct write-back process, where changes made in *RealRisks* are directly inputted into a patient’s chart; (2) a provider-screened write-back process, where changes made in *RealRisks* are brought into a patient’s chart as external data (similarly to a record pulled from another hospital), prompting providers to incorporate changes based on their discretion; and (3) a provider memo, where providers are notified of changes made in *RealRisks* via their in basket or similar notification. Although participants were interested in allowing patients to request edits to their data, they explicitly expressed their discomfort with allowing patients to overwrite existing data (making changes to their EHR without provider or staff approval) (Table 5, Quote 1).

**Table 5.** Participant quotes regarding methods of writing back patient edits to the EHR.

Quote #	Topic	Quote
1	Participants discusses whether patients should be able to overwrite EHR data directly.	*I don’t think direct overwriting by patients is wise. The sophistication and understanding is so variable and even a physician who’s an adult oncologist correcting their child’s vaccine record does not go smoothly. It doesn’t work well to have the consumer update their own medical record without any supervision. Showing a provider a piece of information that they can then review, great. Changing the medical record entry without some kind of validation, not good. I think that’s problematic. I think you should make it easy for the providers to see that the patient recommended the modification or addition of information, like the easier that is, the more likely that the appropriate review or discussion happens, but I would not let it automatically override information. I wouldn’t want the patient’s entry to change actionable medical record items without review. Excerpt P6 (lines 192-196).*
2	IT operations staff discussing preference for external or internal mechanism of data incorporation.	*I would say external…because they’re going through a different platform [RealRisks] to do so…as opposed to the patient reaching out directly to you know the HIM Department to have it updated or they saw the provider at the office visit and noticed that, you know, the doctor said you have a history of so and so, and you go no I’ve never had a history of that. Excerpt P5 (lines 249-258).*
3	Provider discussed how implementing a write-back would impact the current workflow for editing patient data.	*I think patients are really reading their records now. They have access to them. I think before they didn’t have access. So, now since it’s so easy, they really want to make sure that the information is correct, so sometimes even if they’re going to see another provider and they want Columbia records sent to let’s say Mt. Sinai, they want a copy of what’s being sent. So, you know, there may be an initial uptick in the beginning, you know with requests, like changes and stuff, but I think it’s great. Excerpt P5 (lines 262-264).*
4	IT operations staff discussing the legal implications of allowing patient overwriting of EHR data.	*if that breast biopsy result is wrong or you’ve changed it in some way, who would want to add that to our records, and have it used in clinical decision making? Because then, once we put it in the actual medical/legal record, we are medically legal. We have to explain what that is. Why did we make that decision when we have this report from X that says the patient’s biopsy was negative a week ago. Okay, that’s wrong, and it’s a result we didn’t treat and as a result, now the patient’s suing us for all sorts of money. So, you know, you have to understand the bigger picture, which is medical decision-making mistakes are big deals. Excerpt P3 (lines 221-223).*
5	Provider discussing the effect of utilizing existing external data workflow.	*I would want the interaction to be in a structured way, like the care team gets a message that says the patient is requesting updates to these X number of data elements and it then takes you to the module that has the list of these things and list of those things and you can manipulate it to make it more accurate as opposed to a post-it that says, by the way, the patient says this is wrong, go figure out how to put it in the record. Excerpt P1 (lines 225-228).*

Participants across the board felt positive about a write-back method that reintroduced patient data into the EHR, similar to how external data is brought in (Table 5, Quote 2). Considering the likelihood of patient requests to go unaddressed, when asked about the efficacy of utilizing an external request process to reconcile changes with the EHR, a participant suggested that patients’ desire to edit their data would drive the process forward (Table 5, Quote 3).

Participants reiterated that in any implemented write-back, providers should approve requests for changes to medical data within the EHR. Demographic and personal data, however, were stated as reportable to the HIM office or privacy officer for approval. Participants were unsure whether family history information was considered medical or demographic data. Still, they agreed that family history data was unique, and that amendment requests to these fields do not require active verification. A key aspect of the argument for requiring approval of modification requests was grounded in the legal implications of permitting patients to overwrite their information (Table 2, Quote 4). Furthermore, implementing this external data pipeline instead of an in-basket message was stated to provide a more effective workflow for physician approval of changes (Table 2, Quote 5). Participants thus preferred the second of the 3 potential methods of write-back, characterized by provider (and HIM office or privacy officer) approval and a data pipeline similar to that utilized for transferring data from other medical institutions.

### Theme 3: Considerations for application

Although the provider-screened write-back method provides a concrete method through which such a process can be applied into *RealRisks*, the actual implementation of such a write-back is layered and complex, with participants identifying several barriers to technical implementation, workflow, and access. One such concern was the need to inform providers and the HIM office of the maximum amount of information in edit requests to allow easy verification. One provider suggested modifying the *RealRisks* decision aid to encourage uploading test or exam results (Table 6, Quote 1). IT operations staff members concurred (Table 6, Quote 2).

**Table 6.** Participant quotes regarding considerations for applying a write-back.

Quote #	Topic	Quote
1	A provider discussed the benefits of allowing patients to include test results in the write-back mechanism.	*The data is confirmed, because they uploaded some sort of PDF presumably of their result, but you might need a conversation because it changes the course of care because now the information that the clinician is operating under is different. Excerpt P6 (lines 171-172).*
2	IT operations staff discussing the benefits of allowing patients to include test results in the write-back mechanism.	*the physician needs to know how much to trust the data that’s being put to them and if it’s not an emergency, they would more likely than not try to get their own person to do the interpretation through a fresh something…I’m just simply saying that if you put the evidence in there, an uploaded report, then you are doing more. It’s not the patient’s personal opinion; it’s a signed document of a breast biopsy by a pathologist…You can’t cut and paste. You literally have to put the PDF in. Excerpt P8 (lines 300-319).*
3	IT operations staff discussing whether requests to edit names or birth dates should prompt the HIM office to reach out to them or the opposite.	*The second way is the easiest because they have so many requests, they don’t do outpatient, you know, calls to get that information so if we could direct them to the Health Information Management department, that email address, then they could work with them and email them. Excerpt P5 (lines 345-346).*
4	Provider discussing the ideal time in the medical care workflow for patients to interact with a write-back.	*I think, right now in our system the kind of information we’ve been discussing that requires clinician review comes without warning. You know, some of it is in response to there being an upcoming visit and the record actually queries various registries and the patient and asks for information and then the provider hits the chart because there’s a visit and, if they’re feeling diligent, they go through all of these items that are there for their review. But, for something like this, where it is the patient signing up themselves without necessarily involving the clinician, I think it would be pretty difficult to get a doctor to engage with this information passively, meaning that if a patient puts information in here or corrects information, even if the record has a flag saying something has been updated, driving a physician to that record if it is not mandatory that they be there for some reason, reviewing results, seeing a patient, answering a phone call, everybody has too much to do. Excerpt P6 (lines 201-207).*

In addition to providing this medical evidence, IT operations staff also discussed how patients could contact the HIM office. When asked whether *RealRisks* should prompt the HIM office to reach out to patients to change identifying information such as birth dates or names, an IT operations staff member suggested the opposite, stating that patients should be prompted to reach out to the HIM office (Table 6, Quote 3).

Given the heavy caseload handled by the HIM office, ensuring optimal compatibility with existing workflows would require patient-driven communications with the HIM office or privacy officer. Other considerations of workflow mainly focused on providers and the timing with which they would interact with a patient’s data. Although incorporating the write-back into existing data management processes currently utilized for external data would mitigate any vast changes to the way providers reconcile or update charts, providers stressed that the timing of these patient requests would have to be aligned with provider interaction with the specific chart. One provider stated the following:

To address such issues, another provider suggested providing *RealRisks* to patients either within the clinic waiting room itself or in the weeks leading up to an appointment when patients are prompted to update pharmacy and medication information in their patient portal, which would allow both patients and providers to discuss and reconcile discrepancies at the subsequent visit.

## Discussion

We aimed to understand the feasibility of incorporating a write-back process into the *RealRisks* decision aid. Our results suggest that a path exists to develop and implement a write-back method that would not significantly impact existing workflows and would enhance communication between patients and providers. With an understanding of provider and IT operations staff perspectives, future work includes building and piloting a write-back function within *RealRisks* to increase patient ownership and control over their data and to improve the completeness and accuracy of EHR data. As a key function of *RealRisks* is to conduct risk assessment using breast cancer risk prediction models, a more complete integration of the EHR will yield more accurate risk estimates. The lessons from this analysis can also be applied to similar tools in which patients interact with their health data.

Participants emphasized the need for a quality control process for the EHR and agreed that incorporating a write-back function in *RealRisks* would be helpful. However, participants felt uncomfortable with the patients submitting EHR modification requests without provider supervision or discussion. Furthermore, considering who in the patient care workflow would be alerted to an amendment request is key. When considering medical data, alerting all care team members would be beneficial, especially prioritizing nurses and advanced practice providers in the follow-up pool who could approve changes and triage alerts of pertinent medical information to other providers.

To streamline this alert process, an optional submission process appears to be one where patients who modify, correct, or update their health information within *RealRisks* can choose to upload files on recent tests or other medical records and submit them for inclusion into their EHR (contingent upon provider or HIM office or privacy officer’s approval).^12^ As it was suggested that correspondence with the HIM office or privacy officer should be patient-initiated, any relevant patient requests could induce a pop-up/copy-paste of a templated email within the email service upon the user’s device. In interaction with the EHR itself, information written back should be piped into the domains where a provider examining a patient’s chart would find them natively located. For example, mammogram results would be filed under imaging, while other data like lab results might be filed under “flowsheets” rather than solely listed under the “media” tab (the location within EPIC where outside records are displayed). We suggest that participation in this write-back process remain optional for *RealRisks* users, as some patients may want to utilize updated information within *RealRisks* but may have concerns that transferring that information to their EHR may impact their billing or payment processes by impacting their preexisting condition status.

Fundamentally, all health information and EHR management participants are seeking ways to integrate patients more directly and effectively. However, fears about legal implications and the integrity of medical decision-making remain paramount. Implementing a write-back as proposed here would increase patients’ abilities to engage with existing pathways through which edit requests and patient communication occur. Although such measures would not necessarily remove errors or completely address the flaws within the EHR system, they would offer additional pathways for patients, helping them to bypass the high burden of effort resulting from interaction with EHR bureaucracy and increasing the likelihood that their requests are attended to in a timely manner.

One of the most critical questions in assessing implementation feasibility is whether patients or providers would express sufficient buy-in. Given the providers’ strong expression of interest in empowering patients and in the spirit of the 21st Century Cures Act (which establishes patient ownership over their data), provider investment in such a platform is feasible. However, support from IT operations was more tenuous, with respondents maintaining that although such an approach would be effective and important, they were worried about its impact if rolled out incorrectly–especially regarding legal issues and perceptions of increased workload for providers. Furthermore, some difficulty may arise when developing a write-back for an EHR vendor such as EPIC, whose proprietary interests in the EHR platform may raise barriers to accessing the back-end information necessary to smoothly integrate *RealRisks* into the EHR.

## Conclusion

We evaluated the perspectives of providers and IT operations staff on implementing the write-back of PGHD into the EHR. We found that developing such a mechanism was supported by participants who valued it for increasing communication between patients and providers and the accuracy of EHR data. A provider-screened method was supported over a direct write-back method, ensuring that patients would not directly edit or delete aspects of their EHR. Future studies should assess nursing and advanced practice providers along with patient perspectives to complete our understanding of writing back PGHD to the EHR.

### Study limitations

Limitations of this study include the non-generalizability of our findings due to small, purposive sample sizes and the potential for selection bias from volunteer recruitment methods. We also conducted our study in a single academic institution. The sample size (*n* = 9) was limited due to resource constraints. Despite this, we found that thematic saturation in data collected from IT operations staff members was achieved. Although some level of thematic saturation from providers was achieved, additional resources would have expanded the scope of the study to ensure varied perspectives across medical specialties. As discussed above, nurses and advanced practice providers are key practitioners to include in the implementation of an EHR write-back and should be included in subsequent analysis. Additionally, as suggested by the participants in our analysis, including patient perspectives is vital for a complete understanding of the multidirectional nature of health data management and should be a priority for future studies.

## Supplementary Material

ooaf170_Supplementary_Data

## Data Availability

The data analyzed in this article cannot be publicly shared to protect the privacy of participants. However, summaries of the data have been included in the article and Supplementary Materials. Additionally, data will be shared on reasonable request to the corresponding author.

## Supplementary material

Additional quotes are available in Supplementary material is available at *JAMIA Open* online.
