# Supplementary material for: Provider and information technology operations staff perspectives on the feasibility of writing patient-generated health data into the electronic health record
Source: JAMIA Open. 2026 Feb 15;9(1):ooaf170. doi: 10.1093/jamiaopen/ooaf170 (PMC12907014; doi:10.1093/jamiaopen/ooaf170)
Supplement: ooaf170_Supplementary_Data [file ooaf170_supplementary_data.docx]

**Appendix – Supplementary Quote Tables**

**Supplementary Table 1. Participant Quotes Regarding Current Methods of Interaction with Patient Data**

| **Quote #** | **Topic** | **Quote** |
| --- | --- | --- |
| 1 | Provider discussing the mechanism through which patients can access their data. | *Yeah, there’s a patient portal called MyChart or Connect, depending on if you are NYP or Columbia and patients can see large amounts of their data in that context, yeah. Excerpt P1 (line 19).* |
| 2 | IT/operations staff member discussing mechanisms through which patients request physical copies of patient data. | *[patients] can actually sign an online authorization form, or paper form, for a release of information request and we can mail out those records to them, like a paper copy, email, fax or sometimes they ask for the records to be stored on a CD…I would say [patients] mostly [use] Connect. So, they will go onto the patient portal and look for that information.* *Excerpt P5 (lines 33-40).* |
| 3 | Provider discussing methods through which patients request data. | *[Connect] is the only way they can directly access that I know of. I know recently we have a way where you can sort of request the front desk to send patients a letter with their like recent lab results or certain information because we knew not everybody was on the portal, so we’re trying to get better at like sharing data with all of our patients; not just the people on the portal. Otherwise, yeah, I would imagine people can go to like medical records and ask for their data that way. Excerpt P1 (lines 23-27).* |
| 4 | Provider discussing the current workflow for editing patient data. | *A clinician isn’t going to refuse to put that information into the EHR. That’s not going to happen. What happens is, the encounter is closed, your clinic visit has ended, your hospitalization is over, whatever it may be, and you later see your EHR, and your notes and you say oh, this is wrong, didn’t do that. It’s very hard for a clinician to open what’s called an EHR encounter and change the information in there, so you usually have to go to health information management at the hospital and get them to change it, but they’re not the clinician so they don’t want to be changing the clinical data, so you get in this weird loop. So, if it’s during the encounter, it’s usually not a problem, as long as you’re a patient who can advocate for themselves, you know, you can make sure that happens. There can be issues there. It’s when it’s after the encounter and the error is noticed. Excerpt P7 (lines 210-221).* |
| 5 | IT/operations staff member discussing workflow for editing medical data. | *You can see doctor’s notes and lab values and demographics, as you mentioned, yeah, so those are all inputted into the EHR by various different people and staff members. If something’s wrong in a note, and the note was written by a physician, then you would have to actually reach out to that physician to do an amendment to the note. Excerpt P5 (lines 53-54).* |
| 6 | IT/operations staff member discussing patient desire for the ability to apply quality control to their data. | *We’re getting a lot of requests. Patients are reviewing their data and so they’re requesting those patient amendment requests… the reason being is, as you know, we do share information, medical records, through the health system through Epic, so if they sign off on the [Health Information Exchange] form, they want to make sure that the correct information is going to, [other hospitals], so they want to make sure if the information is going back and forth that it is correct. Excerpt P5, lines 50, 57-58.* |
| 7 | IT/operations staff member discussing current patient engagement in editing their data. | *I think that we try to get them to [edit] and review as much as possible. I think the data shows that most patients don’t look to do that, they are more concerned with getting at their clinical data than they are worried about all of the other things…Sure, there are those who are vested in QAing the doctor’s note, which would have a lot of information in it, but yet, most just want to read what the doctor said or share what the doctor said with the next provider or family member or something like that. Excerpt P3 (30-43).* |
| 8 | Provider discussing current issue with data editing workflow. | *the risk is that what the patient requests gets ignored...It sort of sits there perpetually as a request if nobody addresses it. Excerpt P1 (lines 41 and 45).* |

**Supplementary Table 2. Participant Quotes Regarding Methods of Writing Back Patient Edits to the EHR**

| **Quote #** | **Topic** | **Quote** |
| --- | --- | --- |
| 1 | Stakeholder discusses whether patients should be able to overwrite EHR data directly. | *I don’t think direct overwriting by patients is wise. The sophistication and understanding is so variable and even a physician who’s an adult oncologist correcting their child’s vaccine record does not go smoothly. It doesn’t work well to have the consumer update their own medical record without any supervision. Showing a provider a piece of information that they can then review, great. Changing the medical record entry without some kind of validation, not good. I think that’s problematic. I think you should make it easy for the providers to see that the patient recommended the modification or addition of information, like the easier that is, the more likely that the appropriate review or discussion happens, but I would not let it automatically override information. I wouldn’t want the patient’s entry to change actionable medical record items without review. Excerpt P6 (lines 192-196).* |
| 2 | IT/operations staff discussing preference for external or internal mechanism of data incorporation. | *I would say external…because they’re going through a different platform [RealRisks] to do so…as opposed to the patient reaching out directly to you know the HIM Department to have it updated or they saw the provider at the office visit and noticed that, you know, the doctor said you have a history of so and so, and you go no I’ve never had a history of that. Excerpt P5 (lines 249-258).* |
| 3 | Provider discussed how implementing a write-back would impact the current workflow for editing patient data. | *I think patients are really reading their records now. They have access to them. I think before they didn’t have access. So, now since it’s so easy, they really want to make sure that the information is correct, so sometimes even if they’re going to see another provider and they want Columbia records sent to let’s say Mt. Sinai, they want a copy of what’s being sent. So, you know, there may be an initial uptick in the beginning, you know with requests, like changes and stuff, but I think it’s great. Excerpt P5 (lines 262-264).* |
| 4 | IT/operations staff discussing the legal implications of allowing patient overwriting of EHR data. | *if that breast biopsy result is wrong or you’ve changed it in some way, who would want to add that to our records, and have it used in clinical decision making? Because then, once we put it in the actual medical/legal record, we are medically legal. We have to explain what that is. Why did we make that decision when we have this report from X that says the patient’s biopsy was negative a week ago. Okay, that’s wrong, and it’s a result we didn’t treat and as a result, now the patient’s suing us for all sorts of money. So, you know, you have to understand the bigger picture, which is medical decision-making mistakes are big deals. Excerpt P3 (lines 221-223).* |
| 5 | Provider discussing the effect of utilizing existing external data workflow. | *I would want the interaction to be in a structured way, like the care team gets a message that says the patient is requesting updates to these X number of data elements and it then takes you to the module that has the list of these things and list of those things and you can manipulate it to make it more accurate as opposed to a post-it that says, by the way, the patient says this is wrong, go figure out how to put it in the record. Excerpt P1 (lines 225-228).* |

**Supplementary Table 3. Participant Quotes Regarding Considerations for Applying a Write-Back**

| **Quote #** | **Topic** | **Quote** |
| --- | --- | --- |
| 1 | A provider discussed the benefits of allowing patients to include test results in the write-back mechanism. | *The data is confirmed, because they uploaded some sort of PDF presumably of their result, but you might need a conversation because it changes the course of care because now the information that the clinician is operating under is different. Excerpt P6 (lines 171-172).* |
| 2 | IT/operations staff discussing the benefits of allowing patients to include test results in the write-back mechanism. | *the physician needs to know how much to trust the data that’s being put to them and if it’s not an emergency, they would more likely than not try to get their own person to do the interpretation through a fresh something...I’m just simply saying that if you put the evidence in there, an uploaded report, then you are doing more. It’s not the patient’s personal opinion; it’s a signed document of a breast biopsy by a pathologist…You can’t cut and paste. You literally have to put the PDF in. Excerpt P8 (lines 300-319).* |
| 3 | IT/operations staff discussing whether requests to edit names or birth dates should prompt the HIM office to reach out to them or the opposite. | *The second way is the easiest because they have so many requests, they don’t do outpatient, you know, calls to get that information so if we could direct them to the Health Information Management department, that email address, then they could work with them and email them. Excerpt P5 (lines 345-346).* |
| 4 | Provider discussing the ideal time in the medical care workflow for patients to interact with a write-back. | *I think, right now in our system the kind of information we’ve been discussing that requires clinician review comes without warning. You know, some of it is in response to there being an upcoming visit and the record actually queries various registries and the patient and asks for information and then the provider hits the chart because there’s a visit and, if they’re feeling diligent, they go through all of these items that are there for their review. But, for something like this, where it is the patient signing up themselves without necessarily involving the clinician, I think it would be pretty difficult to get a doctor to engage with this information passively, meaning that if a patient puts information in here or corrects information, even if the record has a flag saying something has been updated, driving a physician to that record if it is not mandatory that they be there for some reason, reviewing results, seeing a patient, answering a phone call, everybody has too much to do. Excerpt P6 (lines 201-207).* |
